# Aptamer-functionalized stiff hydrogel for enhanced BMSC enrichment and osteogenesis

**DOI:** 10.1371/journal.pone.0353772

**Published:** 2026-07-16

**Authors:** Jifeng Jing, Fengyu Li, Yu Wang, Shuo Cheng, Lei Guo

**Affiliations:** 1 Department of Orthopedics, Benxi Central Hospital, Benxi, Liaoning, China; 2 Department of Orthopedics, The Third Affiliated Hospital of Jinzhou Medical University, Jinzhou, Liaoning, China; 3 Department of Orthopedics, Liaoning Provincial People’s Hospital, Shenyang, Liaoning, China; 4 Department of Hand Surgery, Shenyang Jingshen Hospital, Shenyang, Liaoning, China; 5 Department of Orthopedics, The First Affiliated Hospital of China Medical University, Shenyang, Liaoning, China; University of Bayreuth, GERMANY

## Abstract

To address the need to both enrich stem cells and direct their osteogenic fate in bone tissue engineering and bone regeneration, we developed a stiffness-gradient hydrogel (~4.5–**33** kPa) functionalized with a cell-enriching aptamer (Apt19s). This design forms a combined “enrich-and-differentiate” system: Apt19s actively enriches endogenous bone marrow-derived mesenchymal stem cells (BMSCs) at the scaffold site, while the osteoinductive high-stiffness niche (~**33** kPa) directs their differentiation. Crucially, the combined cues produced an enhanced osteogenic outcome—evidenced by significantly greater alkaline phosphatase (ALP) activity (>2-fold increase), upregulated RUNX2/osteocalcin (OCN) gene expression (186.5% relative to control), and enhanced mineralization—that surpassed the additive effects of either cue presented independently. This integrated platform provides a practical strategy for developing cell-free osteogenic materials that actively tackle the dual challenges of endogenous cell sourcing and lineage-specific induction.

## 1. Introduction

Bone defect repair requires scaffolds integrating biomechanical guidance and cell enrichment [1]. Hydrogels have been widely used as biomimetic scaffolds due to their tunable mechanical properties and biocompatibility [2,3]. Among various hydrogel materials, methacrylated silk fibroin (SilMA) has emerged as a promising candidate for bone tissue engineering, owing to its excellent biocompatibility, tunable mechanical properties, and high-precision printability [4]. Furthermore, SilMA hydrogels have been shown to support osteogenic differentiation and cartilage formation, making them particularly suitable for osteochondral interface repair [5]. In addition, sodium alginate, a naturally derived polysaccharide, has been widely used in bone tissue engineering due to its excellent biocompatibility, gel-forming ability, and capacity to form composites with ceramics and proteins to mimic the native bone extracellular matrix [6]. Matrix elasticity directs mesenchymal stem cell (MSC) lineage specification: rigid matrices induce osteogenesis [7]. Recent studies have demonstrated that substrate stiffness can regulate stem cell behavior, including adhesion, migration, and differentiation [8–11]. Multilayer scaffolds mimic the osteochondral interface but face complex fabrication and limited cell-binding specificity [12,13]. To enhance specificity, bioactive ligands (e.g., peptides) have been incorporated, improving stem cell attachment [14]. Cell-free, bioactive scaffolds offer a promising strategy; for instance, dopamine-integrated designs enrich cells via immune modulation [2]. Modular scaffolds decouple mechanics and bioactivity [3,15], yet their function remains passive.

To address these limitations, we developed an aptamer-functionalized tunable-stiffness double-network hydrogel that integrates stable cell-binding capacity within a monolithic scaffold. We selected SilMA as the primary matrix material due to its tunable mechanical properties, which enable precise control of stiffness, and proven biocompatibility for supporting MSC adhesion and osteogenic differentiation [4,5]. Aptamers, as short single-stranded oligonucleotides, offer high-affinity targeting and low immunogenicity compared to traditional antibodies [16]. Previous studies have shown that hydrogels with tunable mechanical properties can be fabricated using various techniques, including photopolymerization and diffusion-based crosslinking [17], and such systems have been successfully applied to regulate the differentiation of MSCs [18,19]. The stiffness range was designed with its upper limit falling within the osteogenic range established for matrix-directed MSC differentiation [7], thereby providing a biomimetic mechanical niche. An electrostatically anchored BMSC-specific aptamer (Apt19S) was incorporated to enable autonomous cell enrichment. Apt19S is a single-stranded DNA aptamer specifically selected for binding to rat bone marrow-derived mesenchymal stem cells (BMSCs) with high affinity and specificity. This aptamer was previously reported and validated for targeted BMSC recognition and adhesion in biomaterial scaffolds [20,21]. We hypothesized that this integrated design would combine cell enrichment and osteogenic induction in a single scaffold, wherein aptamer-mediated binding and stiffness-guided osteogenesis enhance stem cell enrichment and osteogenic commitment.

## 2. Materials and methods

Full experimental reagents are listed in S1 Table, and complete synthesis, cell culture, Transwell, qPCR and mineral staining protocols are described in S1–S7 Text.

### 2.1. Synthesis of methacrylated silk fibroin (Silk-MA) and [H NMR spectra were recorded on a Bruker Avance Neo 600 MHz spectrometer at 298 K. Lyophilized Sil-MA powder (approximately 10 mg) was dissolved in 500 μL of D₂O (Sigma-Aldrich, USA) for analysis. Sodium 3-(trimethylsilyl)propionate-2,2,3,3-d₄ (TSP, δ = 0.00 ppm) was used as the internal reference for chemical shift calibration. The zg30 pulse sequence was applied with a spectral width of 20 ppm, 4 scans, and no solvent signal suppression.]H NMR Characterization

Lyophilized SF powder was reacted with glycidyl methacrylate (GMA, Sigma-Aldrich, USA) at 60 °C for 3 h. The reaction mixture was dialyzed (MWCO: 8 kDa) against deionized water for 3 days to remove unreacted GMA and small-molecule byproducts, then lyophilized to obtain purified methacrylated silk fibroin (Sil-MA).

### 2.2. Preparation of SA-Apt19s molecular complex

Sodium alginate (SA, Sigma-Aldrich, USA) was dissolved in 5 mM HEPES buffer (pH 7.4, Gibco, USA) to obtain a 2% (w/v) solution. Amino-modified Apt19s (Sangon Biotech, China) was added to a final concentration of 100 nM and incubated at room temperature for 2 h with gentle agitation. The mixture was dialyzed against PBS (MWCO: 8 kDa) at 4 °C for 24 h to remove unbound aptamer, yielding the SA-Apt19s complex stock solution (~2% w/v SA). A control stock solution was prepared identically without aptamer. Complex formation was verified by FTIR (Nicolet iS10, Thermo Fisher Scientific, USA).

### 2.3. Fabrication of stiffness-gradient hydrogel

The SA-Apt19s stock solution (10 mL) was mixed with Silk-MA (2.000 g) and LAP photoinitiator (0.050 g, Macklin, China) to obtain a precursor solution (final concentrations: SA 2% w/v, Silk-MA 20% w/v, LAP 0.5% w/v). The control precursor was prepared using pure SA. Each solution was injected into a cylindrical mold (diameter: 25 mm) and photocrosslinked under 405 nm UV light (30 mW/cm²) for 5 min. Subsequently, 0.5 M CaCl₂ solution was gently added on top and allowed to diffuse for 2 h at room temperature to obtain hydrogels with tunable stiffness.

For material characterization, three regions were sampled and termed:

S1 (high-stiffness region),

S3 (medium-stiffness region),

S5 (low-stiffness region).

These three regions were selected to represent the full range of mechanical properties of the prepared hydrogels.

### 2.4. Characterization

Chemical structures were analyzed by FTIR and ¹H NMR. Morphology was imaged by SEM (Hitachi SU8010, Japan). Rheological measurements (Discovery HR-2, TA Instruments, USA) were performed to determine storage modulus (G′). Swelling ratios and degradation profiles were assessed in PBS at 37 °C.

### 2.5. In vitro biological evaluation

BMSCs (bone marrow-derived mesenchymal stem cells) were purchased from Procell (Wuhan, China) as primary cells. Cells were cultured in DMEM medium supplemented with 10% fetal bovine serum (FBS) and 1% penicillin-streptomycin at 37 °C in a 5% CO₂ humidified incubator. Cells were used within 3–5 passages to maintain stemness and multipotency, which had been identified and authenticated by the supplier. All in vitro experiments were performed with at least three independent biological replicates. Cell viability was assessed by CCK-8 assay (Dojondo, Japan) at days 1, 3, and 5.

Cell adhesion and enrichment were evaluated using Transwell assays (8 μm pore, Corning, USA). Hydrogels were placed in the lower chamber, and BMSCs were seeded in the upper chamber. Cells attached to or migrated across the membrane were stained with crystal violet after 24 h and quantified using ImageJ (n = 6 per group). Full details of the Transwell assay are provided in Supplementary S4 Text.

Osteogenic differentiation was assessed by ALP activity at days 3, 7, and 14, by qPCR (LightCycler 480, Roche, Switzerland) for RUNX2 and OCN expression at day 14, and by Alizarin Red S staining at day 14. This study did not involve human participants, animal experiments, or the use of primary human tissues. The bone marrow-derived mesenchymal stem cells (BMSCs) were purchased from Procell Life Science & Technology Co., Ltd. (Wuhan, China) with valid ethical certification, and no additional ethical approval was required.

### 2.6. Statistical analysis

All statistical analyses were performed using GraphPad Prism software (Version 9.0). For relative gene expression data (RUNX2, OCN) among different treatment groups (Control, Sil-MA/SA, Sil-MA/SA-Apt19s), a two-way analysis of variance (Two-way ANOVA) was used with “Treatment Group” and “Gene” as fixed factors, followed by Tukey’s honest significant difference (HSD) post hoc test for multiple comparisons. For cell proliferation (CCK-8 assay), cell enrichment (Transwell assay), ALP activity, and matrix mineralization (Alizarin Red S staining) data, statistical significance was determined by one-way or two-way ANOVA followed by Tukey’s post hoc test, as specified in the corresponding result sections. Data are presented as mean ± standard deviation (SD), with ****p < 0.0001, ***p < 0.001, **p < 0.01, and *p < 0.05 considered statistically significant.

## 3. Results

### 3.1. Material synthesis and functionalization

**Fig 1:**
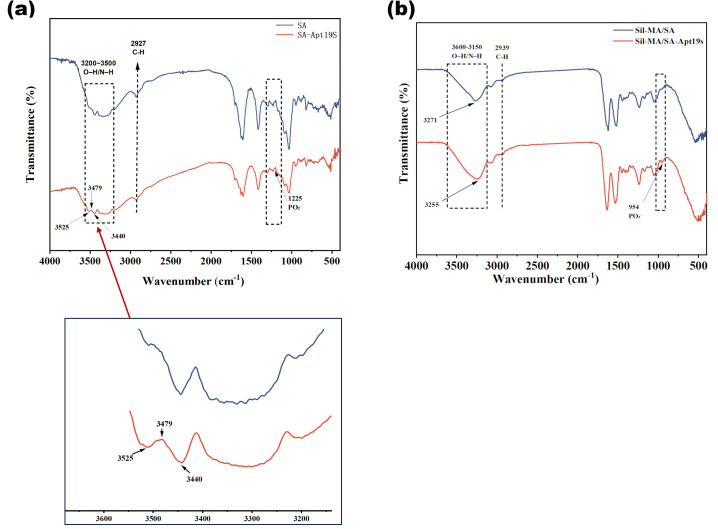
FTIR characterization of SA-Apt19s complex formation and integration. (a) FTIR spectra of pure SA (blue) and SA-Apt19s (red). Inset: Magnified view of the 3200–3600 cm ⁻ ¹ dashed-box region, highlighting the characteristic peaks confirming complex formation. (b) FTIR spectra of Sil-MA/SA and Sil-MA/SA-Apt19s hydrogels, verifying aptamer integration into the dual network.

The successful methacrylation of silk fibroin (SF) was confirmed by ¹H NMR spectroscopy in D₂O (S1 Fig). Compared with pristine SF, new characteristic peaks appeared in the Sil-MA spectrum at δ = 5.66 and 6.09 ppm, which were attributed to the vinyl protons (=CH₂) of the grafted methacryloyl groups. The signal at δ = 1.87 ppm corresponded to the methyl protons (–CH₃) of the methacryloyl moiety. Additionally, the signals at δ = 2.84 and 2.97 ppm were assigned to the modified lysine methylene protons in Sil-MA, which were absent or negligible in the pristine SF spectrum. These new peaks collectively confirm the successful introduction of methacryloyl groups onto the SF backbone.The formation of the SA-Apt19s molecular complex via non-covalent interactions was verified by FTIR (Fig 1a). Compared with pure SA, the SA-Apt19s complex exhibited a new absorption peak at ~1225 cm ⁻ ¹, attributed to PO₂ ⁻ asymmetric stretching of the Apt19s phosphate backbone, confirming the presence of the aptamer. The broadened, rounded peak shape reflects hydrogen-bonding heterogeneity and conformational changes upon complex formation. In the O–H/N–H stretching region (3200–3500 cm ⁻ ¹), the complex displayed a distinctive “W-shaped” fine structure with inflections at ~3525, ~ 3479, and ~3440 cm ⁻ ¹, indicative of hydrogen-bonding network reconstruction—a hallmark of non-covalent enrichment with aptamer-induced local ordering.

The pre-formed SA-Apt19s complex was then integrated into the dual-network hydrogel. FTIR analysis (Fig 1b) confirmed its successful incorporation into the Sil-MA/SA matrix. Compared with the control (Sil-MA/SA), the Sil-MA/SA-Apt19s hydrogel exhibited a new peak at ~954 cm ⁻ ¹ (PO₂ ⁻ symmetric stretching / P–O–C), confirming the presence of the aptamer. In the O–H/N–H stretching region (3600–3150 cm ⁻ ¹, dashed box), the hydrogel containing the complex showed a red-shifted band maximum (from ~3271 to ~3255 cm ⁻ ¹), along with broader, more rounded peak shape and gentler slope—changes characteristic of enhanced hydrogen bonding and structural heterogeneity.

**Fig 2:**
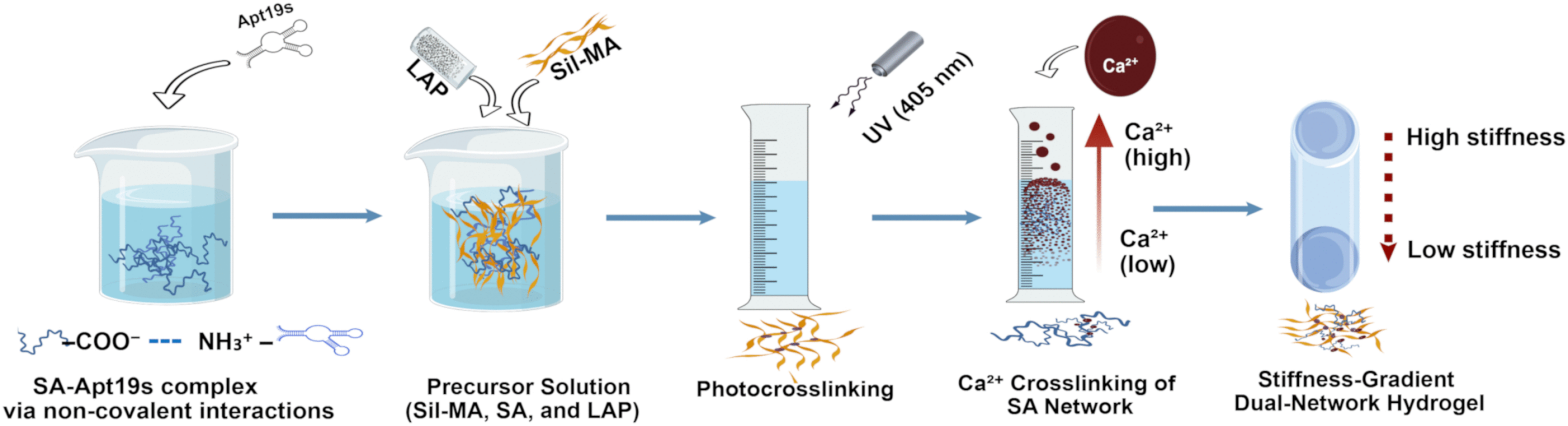
Schematic of dual-network hydrogel fabrication.

Collectively, these results demonstrate that the non-covalent interactions within the SA-Apt19s complex were preserved during photopolymerization and ionic crosslinking, leading to the successful incorporation and stable retention of Apt19s within the dual-network architecture (Fig 2).

### 3.2. Mechanical and structural properties of distinct stiffness regions

**Fig 3:**
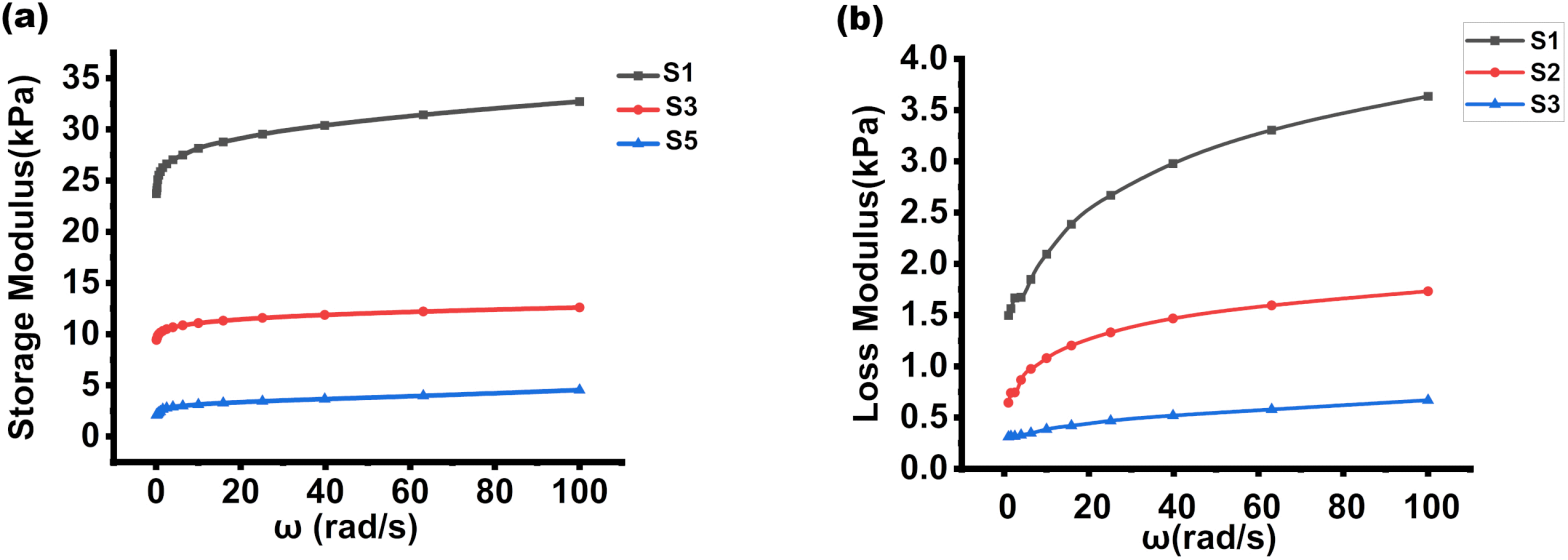
Rheological properties (storage modulus G′ and loss modulus G″) for S1, S3, and S5 regions.

**Fig 4:**
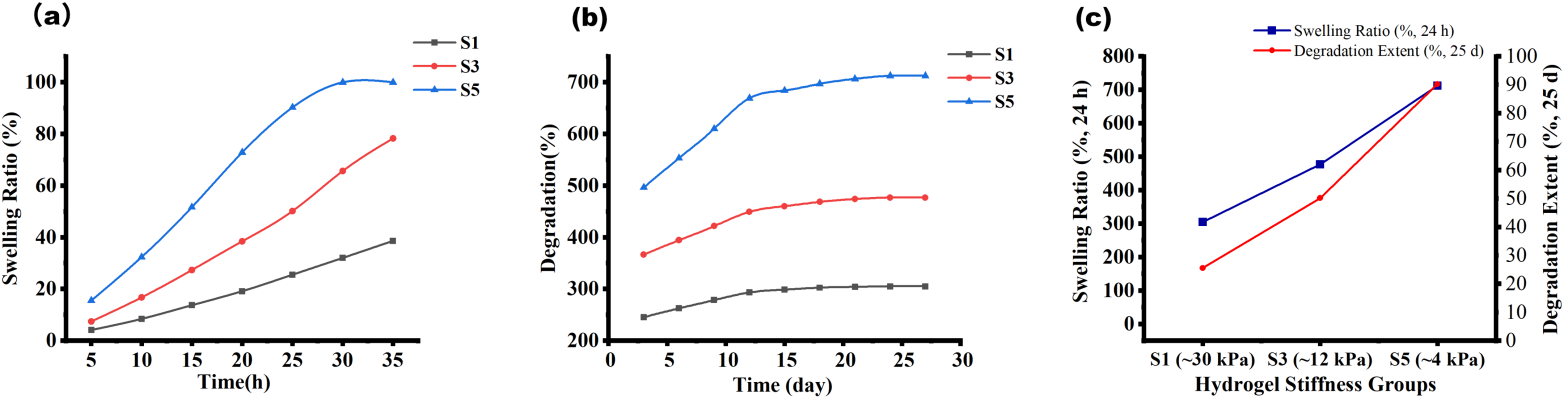
Swelling and degradation of hydrogels with different stiffness. (a) Equilibrium swelling ratio of S1, S3, and S5 hydrogels. (b) In vitro degradation rate of S1, S3, and S5 hydrogels. (c) Relationship between swelling ratio and degradation rate, showing a clear inverse trend.

**Fig 5:**
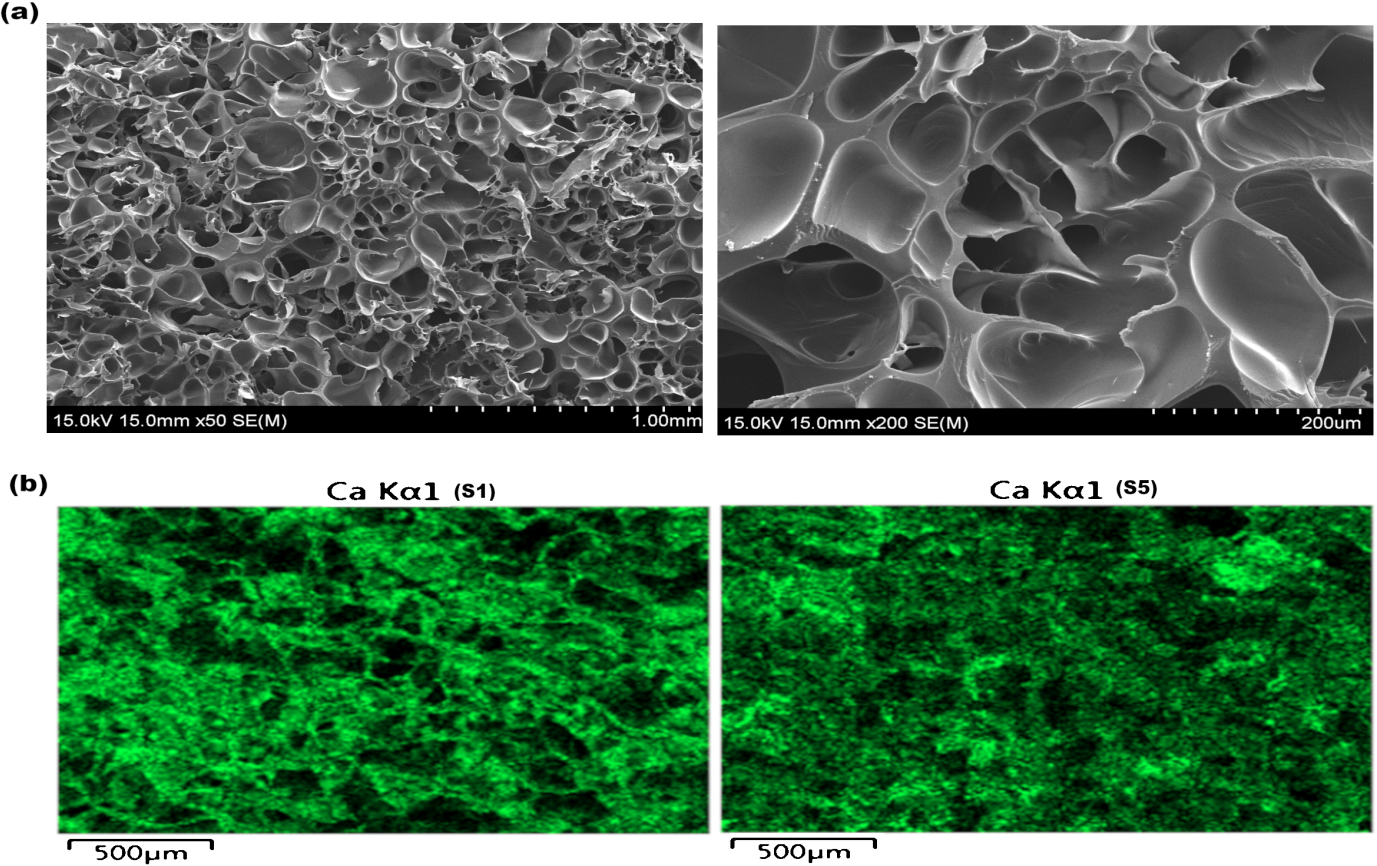
Microstructure and calcium distribution of the hydrogel. (a) SEM images: left panel corresponds to S1 region, right panel corresponds to S5 region, showing the porous microstructure. (b) EDS elemental mapping: calcium distribution in S1 and S5 regions, confirming heterogeneous ionic crosslinking.

For material characterization, three distinct regions were sampled and defined as follows: S1 (high-stiffness region), S3 (medium-stiffness region), and S5 (low-stiffness region). These three regions were selected to represent the full range of mechanical properties within the prepared hydrogels.Rheometry revealed three regions with distinct storage modulus (G′) values: ~ 4.5 kPa (S5, low-stiffness), ~ 18 kPa (S3, medium-stiffness), and ~33 kPa (S1, high-stiffness) at 100 rad/s (Fig 3). This stiffness variation governed the hydrogel’s physicochemical behavior: equilibrium swelling increased from ∼305% in S1 to ∼712% in S5, while in‑vitro degradation showed an inverse trend (Fig 4). The hydrogel exhibits distinct swelling and degradation behaviors related to local crosslinking density. Stiffer regions show lower swelling ratios and slower degradation rates, while less stiff regions display greater water uptake and faster degradation. These differences are directly governed by calcium-mediated ionic crosslinking: higher calcium content leads to stronger crosslinking, limiting water penetration and slowing degradation; lower calcium content results in weaker crosslinking, allowing increased water ingress and accelerating degradation. This trend is consistent with rheological measurements, confirming that local crosslinking strength dominates swelling and degradation properties. SEM images confirmed a homogeneous porous network throughout the hydrogel (Fig 5a). EDS mapping demonstrated a clear spatial difference in Ca² ⁺ concentration, with higher density in the stiff region (S1) and lower density in the soft region (S5) (Fig 5b), directly linking stiffness variation to the underlying ionic crosslink density.

### 3.3. Enhanced BMSC enrichment and proliferation by aptamer incorporation

**Fig 6:**
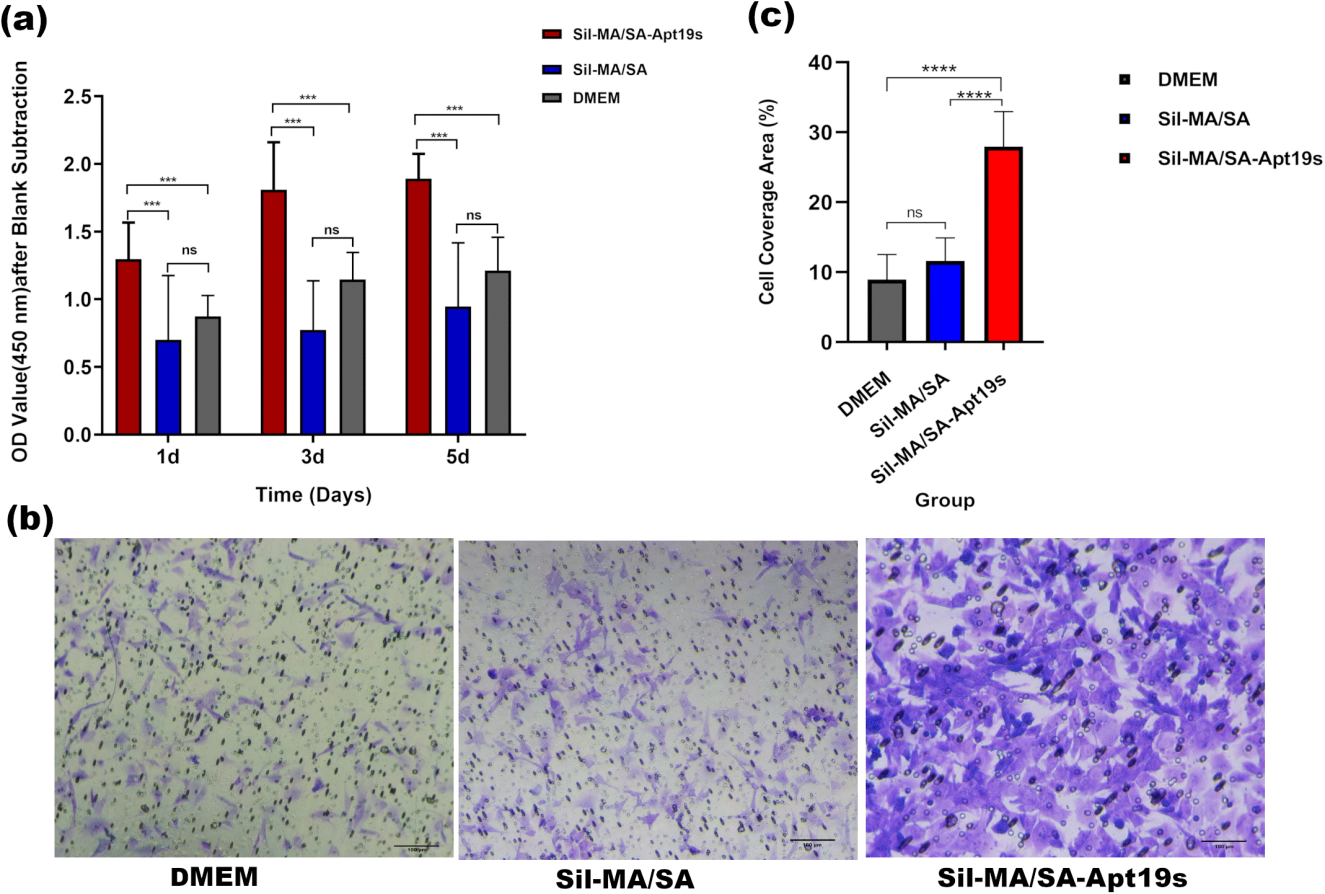
Aptamer-mediated enhancement of BMSC proliferation and enrichment. (a) CCK-8 assay of BMSC proliferation over 5 days on Apt-Gel, Gel, and DMEM control. Data are normalized to day 1 DMEM control and presented as mean ± SD (n = 11 per group). ****p < 0.0001 (two-way ANOVA with Tukey’s post hoc test). (b) Representative crystal violet staining images of migrated BMSCs after 24 h co-culture with Apt-Gel, Gel, or DMEM control. Scale bar = 100 μm. (c) Quantitative analysis of cell adhesion and enrichment expressed as percentage of crystal violet-positive area. Data are presented as mean ± SD (n = 6 per group). ****p < 0.0001 (one-way ANOVA with Tukey’s post hoc test).

The CCK-8 assay confirmed the excellent biocompatibility of the hydrogels and revealed that Apt19s functionalization significantly enhanced BMSC proliferation: Apt-Gel exhibited a significantly higher proliferation rate than both Gel and the DMEM control (Fig 6a).

Transwell assays demonstrated that Apt-Gel significantly enhanced BMSC adhesion and enrichment. (Fig 6b). Quantitative analysis confirmed a greater than 3-fold increase in cell adhesion and enrichment compared to the DMEM control (Fig 6c), underscoring that Apt19s provides a persistent and effective cue for targeted cell homing.

Together, these results establish that Apt19s modification not only promotes stem cell proliferation on the material surface but also actively recruits them, offering a dual strategy for stem cell enrichment.

### 3.4. Combined promotion of osteogenic differentiation

**Fig 7:**
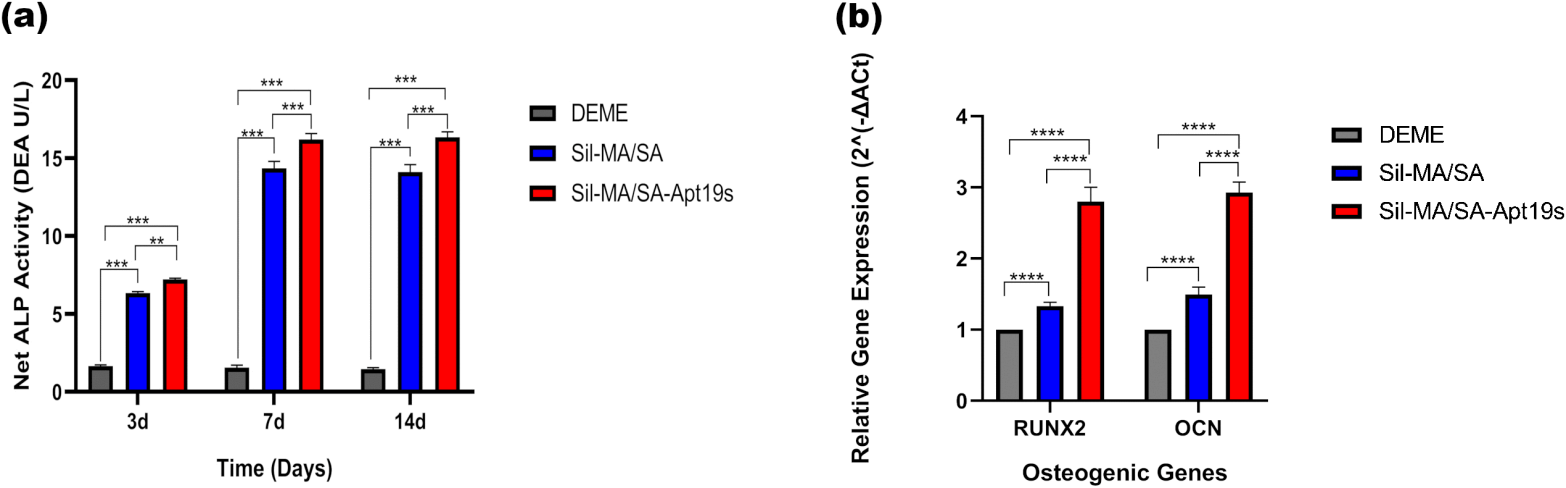
Aptamer functionalization combined with stiff substrate enhances osteogenic differentiation of BMSCs. (a) Alkaline phosphatase (ALP) activity of cells cultured on indicated substrates over time. Data are presented as mean ± SD (n = 3 per group per time point). *p < 0.05, **p < 0.01, ****p < 0.0001 (two-way ANOVA with Tukey’s post hoc test). (b) Relative mRNA expression of osteogenic markers RUNX2 and OCN after 14 days of culture. Expression was normalized to GAPDH and presented relative to the DMEM control. Data are presented as mean ± SD (n = 6 per group). ****p < 0.0001 (one-way ANOVA with Tukey’s post hoc test).

The combined effect of aptamer enrichment and substrate stiffness on osteogenic differentiation was unequivocally demonstrated. First, ALP activity was significantly elevated in cells cultured on both stiff hydrogels compared to the DMEM control (p < 0.0001). Notably, the aptamer-functionalized hydrogel induced significantly higher ALP activity than the stiffness-only hydrogel as early as day 3 (p = 0.0050), with the difference becoming more pronounced at days 7 and 14 (p < 0.0001) (Fig 7a).

At the transcriptional level, the expression of key osteogenic markers RUNX2 and OCN was dramatically upregulated. The aptamer group showed a 186.5% increase relative to the DMEM control, which was significantly greater than the 41.3% increase achieved by stiffness guidance alone (p < 0.0001, Fig 7b).

**Fig 8:**
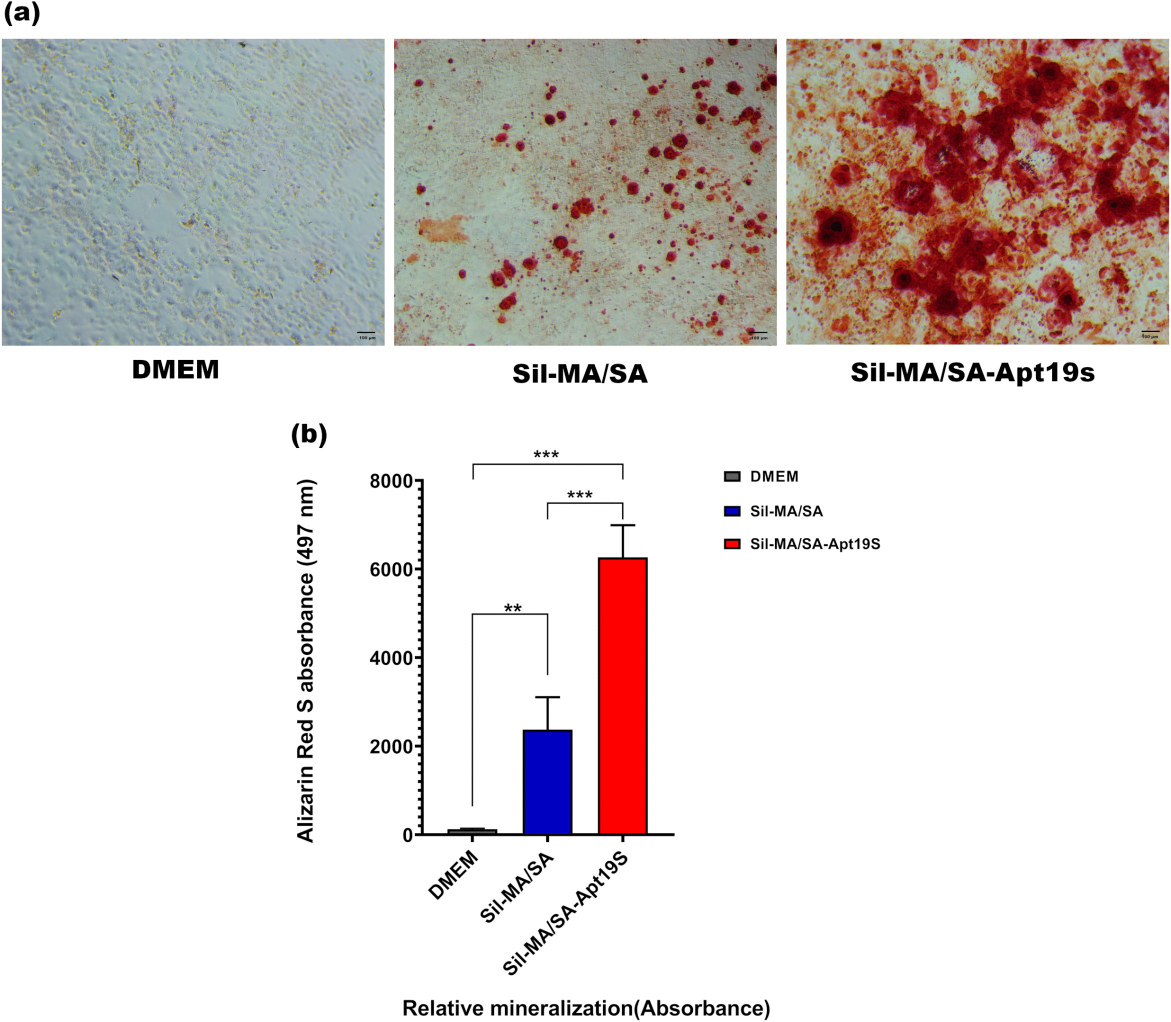
Matrix mineralization assessed by Alizarin Red S staining. (a) Representative images of Alizarin Red S staining. Scale bar = 100 μm. (b) Quantitative analysis of mineralization. Data are presented as mean ± SD (n = 3 per group). ****p < 0.0001 (one-way ANOVA with Tukey’s post hoc test).

Finally, functional mineralization was assessed by Alizarin Red S staining. Representative images showed more intense mineralization on the aptamer-functionalized hydrogels (Fig 8a), and quantitative analysis further confirmed a significant enhancement over both the stiffness-only hydrogel and the DMEM control (p < 0.0001, Fig 8b).

## 4. Discussion

### 4.1. Strategic design of SA-Apt19s complex via non-covalent interactions

A key innovation of this study lies in the incorporation of the BMSC-specific aptamer (Apt19s) into the hydrogel via electrostatic interactions with sodium alginate (SA), rather than conventional physical encapsulation. Physical encapsulation often suffers from burst release and uncontrolled leaching of bioactive molecules, leading to diminished long-term efficacy [22,23]. In contrast, the non-covalent complexation between aminated Apt19s and sodium alginate (SA), driven by the synergistic effect of electrostatic interactions (between -NH₃⁺ of aminated Apt19s and COO⁻ of SA) and hydrogen bonding (between aptamer backbone/amines and SA hydroxyls) as verified by FTIR analysis (Fig 2), is distinct from traditional physical encapsulation or covalent crosslinking and offers three mechanism-based advantages aligned with the core principles of aptamer-functionalized hydrogels [24]: it preserves aptamer conformation and enrichment affinity via mild, site-specific non-covalent interactions (evidenced by the intact PO₂ ⁻ asymmetric stretching peak at ~1225 cm ⁻ ¹ and a “W-shaped” hydrogen-bonding network in the 3200–3500 cm ⁻ ¹ region in FTIR), enables thermodynamically uniform distribution of aptamers throughout the hydrogel matrix by integrating Apt19s as monomolecular or low-oligomer complexes (confirmed by the symmetric, non-split PO₂ ⁻ peak in FTIR, avoiding aggregation issues of physical encapsulation), and achieves sustained, dynamically adjustable presentation of aptamers on the material surface for prolonged cell enrichment—balancing anchoring stability (to prevent aptamer leaching) and conformational flexibility (to facilitate aptamer-target enrichment) while enabling gradual aptamer presentation via moderate SA hydrogel degradation without burst release, consistent with the “sustained molecular recognition” mechanism critical for regenerative medicine.

Notably, this non-covalent anchoring mode was stably retained during the subsequent fabrication of stiffness-gradient hydrogels. As verified by FTIR analysis (Fig 2b), the pre-formed SA-Apt19s complex was successfully integrated into the Sil-MA/SA dual-network matrix: a new peak at ~954 cm ⁻ ¹ (assigned to PO₂ ⁻ symmetric stretching/P–O–C) confirmed the persistent presence of Apt19s, while the red-shifted (from ~3271 to ~3255 cm ⁻ ¹), broader, and more rounded band in the O–H/N–H stretching region (3600–3150 cm ⁻ ¹) reflected enhanced hydrogen bonding and structural heterogeneity—hallmarks of the preserved electrostatic-hydrogen bonding synergistic interaction between Apt19s and SA. This observation further validates the robustness of our non-covalent strategy, which maintains stable aptamer-matrix enrichment even in the complex fabrication process of multi-functional gradient hydrogels.

### 4.2. Tunable-stiffness hydrogel with dual functions for osteogenic induction and BMSC enrichment

The tunable-stiffness hydrogel engineered in this study (∼4.5–33 kPa) exhibits a dual biological function of site-specific osteogenic induction and endogenous bone marrow mesenchymal stem cell (BMSC) enrichment, which fundamentally addresses the core bottleneck of seed cell scarcity in bone tissue engineering and realizes the integration of “stem cell enrichment + directed differentiation” in a single biomaterial scaffold.

The design of the stiffness-tunable system was based on the classic mechanobiological principle that matrix elasticity directs stem cell lineage specification, where Engler et al. first demonstrated that a matrix stiffness of 25–40 kPa is the optimal osteogenic window for mesenchymal stem cells (MSCs) [7]. This foundational principle aligns with Discher et al.’s seminal work establishing that tissue cells actively sense and respond to substrate stiffness through adhesion complexes and the actin-myosin cytoskeleton [25]. Our hydrogel’s high-stiffness region (∼33 kPa) is precisely matched to this osteogenic window, and in vitro characterization confirmed that this stiffness niche can effectively activate the osteogenic differentiation program of BMSCs, upregulating the expression of core osteogenic markers such as Runx2, ALP and OCN—consistent with the findings of Paek et al. [26] that stiffer GelMA hydrogels (0.5–30 kPa) significantly enhance the osteogenic differentiation of human MSCs. BMSCs exhibited stable adhesion, spreading, and proliferative activity across all stiffness regions employed in this study, without explicit dependence on stiffness level for motility.

More importantly, the stiffness-tunable design of the hydrogel is highly compatible with aptamer-mediated BMSC enrichment, supporting coordinated cell enrichment and stiffness-directed osteogenic differentiation. Wang et al. [20] first validated that Apt19s-functionalized silk fibroin scaffolds can specifically recognize and enrich endogenous MSCs in rabbit osteochondral defect models, significantly enhancing cell accumulation at the defect site; Zhou et al. [21] further confirmed that Apt19s-modified 3D-printed GelMA scaffolds effectively promote BMSC adhesion and enrichment in vitro and in vivo. In our system, all stiffness regions support BMSC survival and proliferation, while the high-stiffness region provides a potent osteoinductive niche. This arrangement supports sustained BMSC enrichment and subsequent osteogenic differentiation, avoiding seed cell loss and low differentiation efficiency associated with mismatched cell enrichment and differentiation microenvironments in traditional scaffolds.

In addition, the stiffness range of the hydrogel mimics the native osteochondral interface mechanical microenvironment (from soft cartilage to stiff subchondral bone), which is consistent with the design concept of Liu et al. [27], that biomimetic stiffness-tunable SilMA hydrogels (7–71 kPa) can effectively guide the spatial distribution of cellular responses and tissue repair. Compared with conventional scaffolds that rely on either passive mechanical induction or exogenous cell seeding [28], our dual-function stiffness-tunable hydrogel realizes the in-situ regeneration of bone tissue by enhancing endogenous stem cell enrichment, which not only overcomes the problems of immune rejection, low cell survival rate and complex operation caused by exogenous cell transplantation, but also improves the matching degree of the scaffold with the native tissue mechanical microenvironment through stiffness design, enhancing the functional integration of the regenerated bone tissue with the host tissue.

### 4.3. Potential YAP/TAZ mechanotransduction associated with stiffness-mediated osteogenic differentiation

Matrix stiffness is widely recognized to modulate mesenchymal stem cell fate through the YAP/TAZ mechanotransduction pathway, which converts extracellular mechanical cues into intracellular transcriptional responses [29]. On stiff substrates, enhanced cytoskeletal tension promotes YAP/TAZ nuclear localization [25], while softer environments favor their cytoplasmic retention [29].

In the present system, the high-stiffness region (~33 kPa) supports YAP/TAZ nuclear accumulation [11,29]. Nuclear YAP/TAZ can cooperate with TEAD transcription factors to upregulate osteogenic markers including Runx2, ALP, and OCN [30], and Hadden et al. [31] confirmed that YAP nuclear translocation is tightly associated with matrix stiffness.

Given that direct experimental assessment of YAP/TAZ subcellular localization or activity was not performed, the above associations are based solely on literature precedence and observed stiffness-dependent osteogenic responses. Further studies involving immunofluorescence, western blotting, or gene expression analysis will be required to verify YAP/TAZ activation dynamics and their functional role in osteogenic differentiation.

Mehl et al. [32] reported that lineage-specific YAP/TAZ modulation can enhance osteogenic efficiency while avoiding off-target effects, supporting the potential value of targeted mechanotransduction regulation in bone tissue engineering.

## 5. Conclusion

In summary, we engineered a tunable-stiffness hydrogel with dual functions for BMSC enrichment and osteogenic induction (∼4.5–33kPa), achieved through non-covalent immobilization of the BMSC-specific aptamer (Apt19s) and a biomimetic mechanical niche. This integrated platform supports combined cell enrichment and osteogenic induction: Apt19s mediates over 3-fold enhancement of endogenous BMSC enrichment, while the osteogenic stiffness niche (∼33kPa) drives robust osteogenic differentiation—evidenced by >2-fold higher ALP activity, 186.5% upregulation of RUNX2/OCN expression, and enhanced matrix mineralization compared to control groups. Based on established mechanotransduction mechanisms, we propose this stiffness-dependent osteogenic commitment is mediated by the integrin-RhoA-ROCK-actomyosin cytoskeleton axis regulating YAP/TAZ nuclear-cytoplasmic translocation, which clarifies the molecular link between the hydrogel’s physical properties and biological function. While this provides a compelling rationale for the observed combined effects, direct experimental validation of this pathway within our system—and its crosstalk with aptamer-mediated signaling—remains a key next step. Thus, future work will focus on confirming YAP/TAZ’s role through loss-of-function studies, validating the scaffold’s regenerative capacity in preclinical bone defect models, and further optimizing the platform for clinical translation. Overall, this study delivers a versatile material blueprint for cell-free regenerative biomaterials and advances a design paradigm addressing the dual bottlenecks of endogenous cell sourcing and directed differentiation.

## Supporting information

S1 Table Key reagents and materials used in this study. (DOCX)

S1 Text Aptamer and Primer Sequences. (DOCX)

S2 Text Cell Verification and Ethics Statement. (DOCX)

S3 Text Synthesis and Characterization Methods. (DOCX)

S4 Text Cell Adhesion Assay (Transwell Assay). (DOCX)

S5 Text Alkaline Phosphatase (ALP) Activity Quantification. (DOCX)

S6 Text Quantitative Real-time PCR (qPCR) Analysis for Osteogenic Gene Expression. (DOCX)

S7 Text Alizarin Red S (ARS) Staining for Mineralization Assessment. (DOCX)

S1 Fig Full ¹H NMR spectra of pristine silk fibroin (SF, blue) and methacrylated silk fibroin (Sil-MA, red) recorded at 600 MHz in D₂O at 298 K. The residual H₂O signal of D₂O is labeled at 4.70 ppm. Compared with SF, new characteristic peaks appear in Sil-MA at δ = 6.09 ppm and 5.66 ppm (vinyl protons of methacryloyl group), δ = 2.97 ppm and 2.84 ppm (modified lysine side chains), and δ = 1.87 ppm (methyl protons). These peaks confirm successful methacrylation of SF.
(TIF)
