# Supplementary material for: Aptamer-functionalized stiff hydrogel for enhanced BMSC enrichment and osteogenesis
Source: PLoS One. 2026 Jul 16;21(7):e0353772. doi: 10.1371/journal.pone.0353772 (PMC13374975; doi:10.1371/journal.pone.0353772)
Supplement: S1 Table — (DOCX) [file pone.0353772.s001.docx]

**Table S1. Key reagents and materials used in this study**

| **Reagent/Material** | **Specification** | **Source/Catalog No.** | **Purpose** |
| --- | --- | --- | --- |
| A. Hydrogel Components |  |  |  |
| Sodium Alginate (SA) | Viscosity ≈ 250 mPa·s (1% aqueous solution, 25℃) | Macklin, 180947 | Hydrogel scaffold |
| Silk Fibroin (SF) | Derived from *Bombyx mori* cocoons, molecular weight ≈ 120 kDa | Zhengjing Biotechnology, ZZJ-SF-001 | Methacrylation substrate |
| Glycidyl Methacrylate (GMA) | Contains 100 ppm MEHQ stabilizer | Sigma-Aldrich, 77915-25ML | Methacrylation reagent |
| 1-(3-Dimethylaminopropyl)-3-ethylcarbodiimide Hydrochloride (EDC·HCl) | ≥98.5% | Aladdin, E106863 | Carboxyl activation |
| N-Hydroxysuccinimide (NHS) | ≥98% | Aladdin, H106363 | Carboxyl activation |
| Lithium Phenyl-2,4,6-Trimethylbenzoylphosphinate (LAP) | ≥98% | Macklin, L105405 | Photoinitiator |
| Calcium Chloride (CaCl₂) | Anhydrous, ≥96% | Sinopharm Group, 20011160 | Ionic crosslinker |
| B. Aptamer Reagents |  |  |  |
| Apt19S (Amino-modified) | HPLC purity ≥ 98%, 5′-NH₂-(A)₉-[Specific Sequence]-3′ | Sangon Biotechnology, Custom Synthesis | BMSC enrichment ligand |
| Apt19S (FAM-labeled) | HPLC purity ≥ 98%, 5′-[Specific Sequence]-FAM-3′ | Sangon Biotechnology, Custom Synthesis | Fluorescent tracing |
| C. Cell Culture |  |  |  |
| Rat Bone Marrow Mesenchymal Stem Cells (BMSCs) | C57BL/6 strain, passages 3-5 | Procell, CP-M129 | *In vitro* experiments |
| Low Glucose DMEM Medium | Contains L-glutamine | Gibco, C11995500BT | Cell culture medium |
| Fetal Bovine Serum (FBS) | Qualified grade, South American origin | Gibco, 10099141C | Medium supplement |
| Penicillin-Streptomycin Double Antibiotic | 10,000 U/mL | Gibco, 15140122 | Antibiotic |
| D. Molecular Biology |  |  |  |
| TRIzol Reagent | - | Invitrogen, 15596026 | RNA extraction |
| PrimeScript RT reagent Kit | Contains gDNA Eraser | Takara, RR047A | cDNA synthesis |
| TB Green Premix Ex Taq II | - | Takara, RR820A | Quantitative Real-Time PCR (qPCR) |
| CCK-8 Assay Kit | - | Dojindo, CK04 | Cell viability detection |
