# Supplementary material for: Aptamer-functionalized stiff hydrogel for enhanced BMSC enrichment and osteogenesis
Source: PLoS One. 2026 Jul 16;21(7):e0353772. doi: 10.1371/journal.pone.0353772 (PMC13374975; doi:10.1371/journal.pone.0353772)
Supplement: S1 Text — (DOCX) [file pone.0353772.s002.docx]

**S1 Text. Aptamer and Primer Sequences**

*S1.1 Aptamer Apt19S Sequences*

All aptamers were synthesized and purified by Sangon Biotechnology (Shanghai) Co., Ltd. (HPLC C-18 column, purity ≥ 98%).

| **Aptamer Type** | **Sequence (5′→3′)** |
| --- | --- |
| Apt19S (Unmodified) | AGGTCAGATGAGGAGGGGGACTTAGGACTGGGTTTATGACCTATGCGTG |
| Amino-modified Apt19S | NH₂-(A)₉-AGGTCAGATGAGGAGGGGGACTTAGGACTGGGTTTATGACCTATGCGTG |
| FAM-labeled Apt19S | AGGTCAGATGAGGAGGGGGACTTAGGACTGGGTTTATGACCTATGCGTG-FAM |

Note: The (A)₉ polyadenylic acid spacer facilitates aptamer display and reduces steric hindrance during binding to BMSC surface receptors.

*S1.2 qPCR Primer Sequences*

Primer sequences were designed based on existing literature and verified for specificity via melt curve analysis (single peak) and standard curve validation (amplification efficiency: 95%-105%, R² > 0.99) (S3 Fig). All validation experiments were performed with n = 3 independent biological replicates, each with 3 technical replicates.

| **Gene** | **Forward Primer (5′→3′)** | **Reverse Primer (5′→3′)** | **Accession Number** | **Note** |
| --- | --- | --- | --- | --- |
| RUNX2 | GCCGGGAATGATGAGAACTA | GGTGAAACTCTTGCCTCGTC | BC108919.1 | Key osteogenic transcription factor |
| OCN | GCCATCACCCTGTCTCCTAA | GCTGTGGAGAAGACACACACG | BC113432.1 | Late osteogenic marker |
| GAPDH | CATCCCAGAGCTGAACC | CTGGTCCTCAGTGTAGC | AF261085.1 | Housekeeping gene (internal control) |

*Reference for primer design: Expression of RUNX2/LAPTM5 during mineralization induction of MC3T3-e1 cells and its possible relationship with autophagy. *
