# Supplementary material for: Aptamer-functionalized stiff hydrogel for enhanced BMSC enrichment and osteogenesis
Source: PLoS One. 2026 Jul 16;21(7):e0353772. doi: 10.1371/journal.pone.0353772 (PMC13374975; doi:10.1371/journal.pone.0353772)
Supplement: S2 Text — (DOCX) [file pone.0353772.s003.docx]

# **S2 Text. Cell Verification and Ethics Statement**

## *S2.1 Cell Source Specification*

The rat bone marrow-derived mesenchymal stem cells (BMSCs) used in this study were commercially obtained with the following details:

Supplier: Procell Life Science & Technology Co., Ltd. (Wuhan, China)

Product Name: Rat Bone Marrow Mesenchymal Stem Cells

Catalog No.: CP-M129

Specification: 5×10⁵ Cells per T25 Flask (Ambient Temperature Shipping)

Passage Usage: Cells from passages 3 to 5 were utilized for all experiments (to ensure cell purity and stemness)

## *S2.2 Ethics Statement*

This cell line is a commercially available and ethically sourced product. Its procurement and experimental use comply with the supplier's stated ethical and quality assurance guidelines. No animal isolation, sacrifice, or in vivo experimental procedures were performed in this study. Therefore, no additional approval from an Institutional Animal Care and Use Committee (IACUC) or equivalent ethical review body was required.

## *S2.3 Cell Identity Verification*

According to the minimal criteria defined by the International Society for Cell Therapy (ISCT) for mesenchymal stromal cells, cell identity was confirmed by flow cytometry analysis of characteristic surface markers. The analysis was performed with three independent biological replicates (n = 3), each comprising three technical replicates.
