# Supplementary material for: Aptamer-functionalized stiff hydrogel for enhanced BMSC enrichment and osteogenesis
Source: PLoS One. 2026 Jul 16;21(7):e0353772. doi: 10.1371/journal.pone.0353772 (PMC13374975; doi:10.1371/journal.pone.0353772)
Supplement: S3 Text — (DOCX) [file pone.0353772.s004.docx]

# **S3 Text. Synthesis and Characterization Methods**

## *S3.1 Synthesis of Methacrylated Silk Fibroin (Silk-MA)*

For each independent synthesis batch (n = 3), 1 g of soluble lyophilized silk fibroin and 5 g of sodium bromide (NaBr) were dissolved in 100 mL of deionized water and stirred at 60°C for 3 h until completely dissolved. Subsequently, 500 μL of glycidyl methacrylate (GMA) was injected into the solution, and the reaction was continued at 60°C for 3 h. After the reaction, the solution was transferred to dialysis bags (molecular weight cutoff, MWCO: 3.5 kDa) and dialyzed against deionized water for 3 days (with frequent water changes to remove unreacted GMA). The purified solutions from the three independent batches were combined, frozen, and lyophilized for 48 h to obtain Silk-MA powder for subsequent experiments.

## *S3.2 Preparation of Sil-MA/SA-Apt19s Biomimetic Stiffness-Gradient Hydrogel*

- Precursor Solution Preparation

Photoinitiator Solution: A 0.5% (w/v) solution of lithium phenyl-2,4,6-trimethylbenzoylphosphinate (LAP) was prepared in PBS (pH 7.4) and stored protected from light. Preparation was performed with n = 3 independent biological replicates.

Aptamer Stock Solution: Amino-modified Apt19s lyophilized powder was reconstituted in sterile PBS to 1 mM as a stock solution, aliquoted, and stored at -20°C. Preparation was performed with n = 3 independent biological replicates.

Polymer Dissolution and Aptamer Incorporation: Precisely weighed Silk-MA and SA powders were dissolved to final concentrations of 20% (w/v) and 2% (w/v), respectively, in 0.5% LAP solution. The mixture was vortexed briefly and stirred magnetically at room temperature (light-protected) for ≥30 min until a homogeneous, particle-free viscous solution was obtained. An appropriate volume of 1 mM Apt19s stock was added to achieve a final concentration of 100 nM (Note 1), and stirring was continued for 10 min to ensure uniform dispersion. For the control group (Sil-MA/SA without aptamer), an equal volume of sterile PBS was added instead of aptamer stock. All precursor solutions were kept on ice or at 4°C and used within 2 h of preparation. Preparation was performed with n = 3 independent biological replicates.

Note 1: Adding Apt19s at the precursor stage (prior to crosslinking) ensures its stable incorporation within the hydrogel network during photopolymerization and ionic crosslinking.

- Fabrication of Stiffness-Gradient Hydrogel

Optional Purification: Precursor solution was transferred to a dialysis tube (MWCO 3.5 kDa) and dialyzed against PBS at 4°C for 12 h (buffer changed every 4-6 h) to remove unreacted species. Purification was performed with n = 3 independent biological replicates.

Photocrosslinking (First Network): The solution was injected into a cylindrical mold (diameter: 25 mm) and exposed to 405 nm UV light (intensity: 30 mW/cm²) for 5 min to form the primary covalently crosslinked Silk-MA network. Photocrosslinking was performed with n = 3 independent biological replicates.

Gradient Ionic Crosslinking (Second Network): 0.5 M CaCl₂ solution was carefully poured over the photocrosslinked gel in the mold and left undisturbed at room temperature for 2 h. Ca²⁺ diffusion from top to bottom created a concentration gradient, leading to spatially varying ionic crosslinking of the SA network and establishing a stiffness gradient (highest at the top). Crosslinking was performed with n = 3 independent biological replicates.

Post-processing: The gradient hydrogel was removed, rinsed thoroughly with PBS, and stored in PBS at 4°C until use.

## *S3.3 Rheological and Microstructural Characterization*

All characterization experiments were performed with n = 3 independent biological replicates, each including 3 technical replicates (repeated detections of the same biological sample).

(I) Rheological Properties and Stiffness Gradient Characterization

Rheological tests were performed using a strain-controlled rotational rheometer (TA Instruments HR-10) equipped with a 25 mm parallel plate fixture and Peltier temperature control system (25°C). Data processing was performed with Origin software (Version 2023b), and statistical analysis with GraphPad Prism (Version 9.5.1).

To quantify the stiffness gradient, equilibrium-swollen hydrogels were cut into five equal-thickness slices perpendicular to Ca²⁺ diffusion direction (numbered S1 to S5). S1 = closest to Ca²⁺ source (high stiffness), S5 = farthest (low stiffness). S1, S3, and S5 were selected as representative regions for detailed analysis.

During testing, sample slices were placed in the center of the lower fixture, the upper fixture was lowered to a 1600 μm gap, and excess material was trimmed. Within the linear viscoelastic region (LVR), a fixed oscillatory strain (0.2%) was applied for frequency sweep (0.1-100 rad/s). The instrument recorded storage modulus (G′), loss modulus (G″), loss factor (tan δ = G″/G′), complex viscosity (η*), and other parameters.

Stiffness gradients were quantified by comparing G′ values of S1, S3, and S5 at 1 Hz (ω = 6.28 rad/s). Data are expressed as mean ± standard deviation (n = 3 independent experiments, 3 technical replicates each).

(II) SEM and EDS Analysis

Lyophilized hydrogel samples were soaked in liquid nitrogen for 2 min and fractured to obtain fresh cross-sections. Samples were fixed on sample stages with conductive adhesive and coated with a 10 nm gold/palladium alloy via ion sputtering (Quorum Q150T ES) to enhance conductivity.

SEM Observation: A Hitachi SU8010 field emission SEM was used to observe cross-sectional morphology. Imaging parameters: 5.0 kV accelerating voltage, 15.0 mm working distance, ×50 magnification (secondary electron mode) for overview (≈1 mm field of view); 15.0 kV, ×200 magnification for fine details (≈200 μm field of view).

EDS Analysis: An Oxford X-MaxN 50 mm² spectrometer was used for area scans to analyze Ca²⁺ distribution. Semi-quantitative analysis was based on characteristic peaks of C, O, Ca, and Cl (results expressed as weight percentage, wt%). At least three distinct sites were analyzed per region (S1/S5) (n = 3 independent biological replicates).

## *S3.4 Fourier Transform Infrared Spectroscopy (FTIR)*

Lyophilized Sil-MA/SA and Sil-MA/SA-Apt19s hydrogels were ground into fine powder, mixed with dry KBr (1:100 mass ratio), ground in an agate mortar, and pressed into transparent thin slices. A Nicolet iS50 FTIR spectrometer was used for transmission mode scanning (4000-500 cm⁻¹, resolution 4 cm⁻¹, 32 scans) to obtain high signal-to-noise ratio spectra. All experiments were performed with n = 3 independent biological replicates, and data processed with Origin software (Version 2023b).

## *S3.5 Proton Nuclear Magnetic Resonance Spectroscopy (¹H NMR)*

A Bruker AVANCE III 400 MHz NMR spectrometer was used (D₂O as solvent, 25°C). Parameters: 64 scans, 2 s relaxation delay, 20 ppm scan width. All experiments were performed with n = 3 independent biological replicates, and data processed with Origin software (Version 2023b).

## *S3.6 Swelling and Degradation Performance Tests*

### *S3.6.1 Experimental Methods*

Hydrogels were divided into three representative regions (S1: high stiffness, S3: medium stiffness, S5: low stiffness). Five parallel samples were set per region (n = 5 independent biological replicates, 3 technical replicates each), and initial dry weight (W₀) was measured after lyophilization. Data processing was performed with Origin software (Version 2023b), and statistical analysis with GraphPad Prism (Version 9.5.1).

Swelling Test: Samples were immersed in 5 mL PBS (pH 7.4) at 37°C. At predetermined time points (0, 3, 6, 9, 12, 15, 18, 21, 24, 27 h), samples were removed, surface moisture was blotted with filter paper, and wet weight (Wₛ) was measured. Swelling ratio (SR) was calculated as:

SR (%) = [(Wₛ − W₀) / W₀] × 100%

Degradation Test: Samples were immersed in 5 mL PBS at 37°C (buffer changed every 3 days). At predetermined time points (5, 10, 15, 20, 25, 30, 35 days), samples were rinsed with deionized water, lyophilized, and residual dry weight (W_d) was measured. Degradation percentage (PD) was calculated as:

PD (%) = [(W₀ − W_d) / W₀] × 100%

### *S3.6.2 Key Results*

Swelling and degradation behaviors showed significant stiffness dependence. Tables S2 and S3 summarize data at key time points (mean ± standard deviation, n = 5 independent samples per region, 3 technical replicates each).

Table S2. Swelling kinetics of representative gradient hydrogel regions (S1, S3, S5)

| **Time (h)** | **S1 (High Stiffness)** | **S3 (Medium Stiffness)** | **S5 (Low Stiffness)** |
| --- | --- | --- | --- |
| 3 | 245.7 ± 1.7 | 367.0 ± 1.7 | 496.6 ± 1.7 |
| 6 | 262.8 ± 1.6 | 394.5 ± 1.6 | 553.4 ± 1.6 |
| 9 | 278.7 ± 1.6 | 422.0 ± 1.6 | 610.2 ± 1.6 |
| 12 | 293.6 ± 1.5 | 449.5 ± 1.5 | 669.3 ± 1.5 |
| 15 | 299.0 ± 1.5 | 460.4 ± 1.5 | 684.1 ± 1.5 |
| 18 | 302.7 ± 1.5 | 468.7 ± 1.5 | 697.2 ± 1.5 |
| 21 | 304.3 ± 1.5 | 474.2 ± 1.5 | 706.8 ± 1.5 |
| 24 | 305.3 ± 1.5 | 477.0 ± 1.5 | 712.5 ± 1.5 |
| 27 | 305.3 ± 1.5 | 477.0 ± 1.5 | 712.5 ± 1.5 |

Table S3. Degradation kinetics of representative gradient hydrogel regions (S1, S3, S5)

| **Time (day)** | **S1 (High Stiffness)** | **S3 (Medium Stiffness)** | **S5 (Low Stiffness)** |
| --- | --- | --- | --- |
| 5 | 4.2 ± 0.7 | 7.5 ± 0.9 | 15.6 ± 1.1 |
| 10 | 8.5 ± 0.8 | 16.8 ± 1.0 | 32.4 ± 1.2 |
| 15 | 13.8 ± 0.9 | 27.4 ± 1.1 | 51.7 ± 1.3 |
| 20 | 19.2 ± 1.0 | 38.5 ± 1.2 | 72.9 ± 1.4 |
| 25 | 25.6 ± 1.1 | 50.2 ± 1.3 | 90.3 ± 1.4 |
| 30 | 32.1 ± 1.2 | 65.7 ± 1.4 | 100.0 ± 0.0 |
| 35 | 38.7 ± 1.3 | 78.3 ± 1.5 | 100.0 ± 0.0 |
