# Supplementary material for: Aptamer-functionalized stiff hydrogel for enhanced BMSC enrichment and osteogenesis
Source: PLoS One. 2026 Jul 16;21(7):e0353772. doi: 10.1371/journal.pone.0353772 (PMC13374975; doi:10.1371/journal.pone.0353772)
Supplement: S4 Text — (DOCX) [file pone.0353772.s005.docx]

# **S4 Text. Cell Adhesion and Enrichment Assay**

## *S4.1 Experimental Purpose*

To evaluate the enrichment capability of Apt19S-functionalized tunable-stiffness hydrogels (Sil-MA/SA-Apt19s) towards rat BMSCs, compared to unmodified tunable-stiffness hydrogels (Sil-MA/SA) and a blank control (serum-free DMEM). All experiments were performed with n = 3 independent biological replicates.

## *S4.2 Experimental Materials and Instruments*

Cells: Rat BMSCs (C57BL/6 strain), passages 3-5 (Procell, CP-M129)

Hydrogel Samples: Sil-MA/SA-Apt19s and Sil-MA/SA hydrogels (prepared as per S3 Text), cut into circular discs (diameter: 12 mm, thickness: 2 mm) and sterilized by UV irradiation (30 min, 15 min per side)

Medium:
a) Complete Medium: Low Glucose DMEM (Gibco, C11995500BT) + 10% FBS (Gibco, 10099141C) + 1% Penicillin-Streptomycin (Gibco, 15140122)
b) Starvation Medium: Serum-free Low Glucose DMEM

Transwell Inserts: 24-well format, 8 μm pore size (Corning, 3422)

Staining Reagents: 0.1% Crystal Violet Staining Solution (Solarbio, G1063); 70% ethanol; PBS (pH 7.4)

Instruments: CO₂ Incubator (37°C, 5% CO₂, Thermo Scientific, 3111); Inverted Microscope (Olympus IX73); ImageJ Software (Version 1.54f); Centrifuge (Eppendorf, 5810R); Ultra-clean Bench (SuZhou Antai Air Technology, SW-CJ-2FD)

## *S4.3 Experimental Methods*

Cell Preparation: BMSCs were cultured in complete medium to 80-90% confluence. Cells were washed with PBS, detached with 0.25% trypsin-EDTA (Gibco, 25200056), neutralized with complete medium, and centrifuged (1000 rpm, 5 min). The pellet was resuspended in starvation medium, and cell concentration was adjusted to 5 × 10⁴ cells/mL.

Group Setting and Lower Chamber Pre-incubation: Three groups were set (3 technical replicates per independent experiment):

Experimental Group: Lower chamber with Sil-MA/SA-Apt19s hydrogel + 600 μL starvation medium

Control Group: Lower chamber with Sil-MA/SA hydrogel + 600 μL starvation medium

Blank Control Group: Lower chamber with 600 μL starvation medium only

The 24-well plate was placed in the incubator for 1 h to allow chemoattractant diffusion and environmental equilibration.

Transwell Assay Setup: A Transwell insert was placed into each lower chamber. 100 μL BMSC suspension (5 × 10³ cells) was added to the upper chamber. The plate was returned to the incubator for 24 h to allow cell migration.

Cell Staining: Non-migrated cells on the upper membrane surface were removed with a cotton swab. Migrated cells on the lower surface were fixed with 70% ethanol and stained with 0.1% crystal violet for 15 min, then rinsed with PBS.

Image Acquisition and Quantitative Analysis: The entire polycarbonate membrane was imaged with an inverted microscope. Crystal violet-positive area percentage (relative to total membrane area) was quantified with ImageJ software (consistent threshold applied to all images). Data from 3 independent experiments (2 technical replicates analyzed per experiment) were pooled (n = 6 membranes per group for statistical comparison).

## *S4.4 Statistical Analysis*

Quantitative data are presented as mean ± standard deviation (SD). Statistical significance among groups was determined by one-way ANOVA followed by Tukey's post hoc test (GraphPad Prism software). P < 0.05 was considered statistically significant.
