# Supplementary material for: Aptamer-functionalized stiff hydrogel for enhanced BMSC enrichment and osteogenesis
Source: PLoS One. 2026 Jul 16;21(7):e0353772. doi: 10.1371/journal.pone.0353772 (PMC13374975; doi:10.1371/journal.pone.0353772)
Supplement: S5 Text — (DOCX) [file pone.0353772.s006.docx]

# **S5 Text. Alkaline Phosphatase (ALP) Activity Quantification**

## *S5.1 Purpose*

To quantitatively assess the early osteogenic differentiation potential of rat BMSCs cultured on different hydrogel substrates by measuring ALP activity at days 3, 7, and 14.

## *S5.2 Materials and Reagents*

- ALP Assay Kit: Alkaline Phosphatase Assay Kit (Colorimetric, Beyotime, P0321M)
- Cell Lysis Buffer: Western and IP Cell Lysis Buffer (without inhibitors, Beyotime, P0013J)
- Microplate Reader: Multi-mode microplate reader (BioTek Synergy H1)
- BCA Protein Assay Kit: Pierce™ BCA Protein Assay Kit (Thermo Scientific, 23225)
- Other: PBS (pH 7.4), 96-well clear flat-bottom microplates, centrifuge, ice bath

## *S5.3 Experimental Procedure*

All steps were performed with n = 3 independent biological experiments (3 technical replicates per experiment).

1. Sample Collection and Lysis: At days 3, 7, and 14 post-osteogenic induction, culture medium was aspirated. Cells were washed twice with ice-cold PBS. 200 µL ice-cold lysis buffer was added to each sample, incubated on ice for 15 min (intermittent vortexing), and centrifuged at 12,000 × g for 15 min at 4°C. Supernatants (cell lysates) were collected and kept on ice for immediate assay or stored at -80°C.
2. Total Protein Quantification: Total protein concentration was determined with the BCA Protein Assay Kit. A BSA standard curve was prepared, and absorbance was measured at 562 nm to calculate protein concentration (µg/µL).
3. ALP Activity Measurement (per manufacturer's protocol for Beyotime P0321M kit):

a) Reagent Preparation: Chromogenic substrate was dissolved in 5 mL Assay Buffer (working solution, ice-protected from light, used within 6 h). 0.5 mM p-nitrophenol (pNP) standard working solution was prepared by diluting 10 µL 10 mM pNP stock with 190 µL Assay Buffer.

b) Standard Curve and Reaction Setup: Reactions were set up in 96-well plates:

- - Standard Wells: 0, 4, 8, 16, 24, 32, 40 µL 0.5 mM pNP + Assay Buffer to 100 µL (corresponding to 0, 2, 4, 8, 12, 16, 20 nmol pNP)
  - Sample Wells: 50 µL cell lysate + 50 µL substrate working solution
  - Sample Background Control: 50 µL cell lysate + 50 µL Assay Buffer (no substrate)

c) Incubation and Termination: Plates were incubated at 37°C for 30 min, then 100 µL Stop Solution was added to each well.

1. **Absorbance Measurement and Calculation:** Absorbance of pNP was measured at 405 nm. A standard curve was plotted (pNP amount vs. absorbance) (**S2 Fig**), and pNP production in samples was calculated. ALP activity was defined as 1 U = 1 µmol pNP produced per minute under assay conditions (pH 9.8, 37°C). Specific ALP activity was calculated as:

ALP Activity (U/µg protein) = [pNP_sample (nmol) × 10⁻³] / [Incubation Time (min) × Total Protein in Reaction (µg)]

## *S5.4 Statistical Analysis*

Data are presented as mean ± SD (n = 3 independent biological experiments). Statistical comparisons among groups were performed by one-way ANOVA followed by Tukey's post hoc test (GraphPad Prism 9.0). P < 0.05 was considered statistically significant.


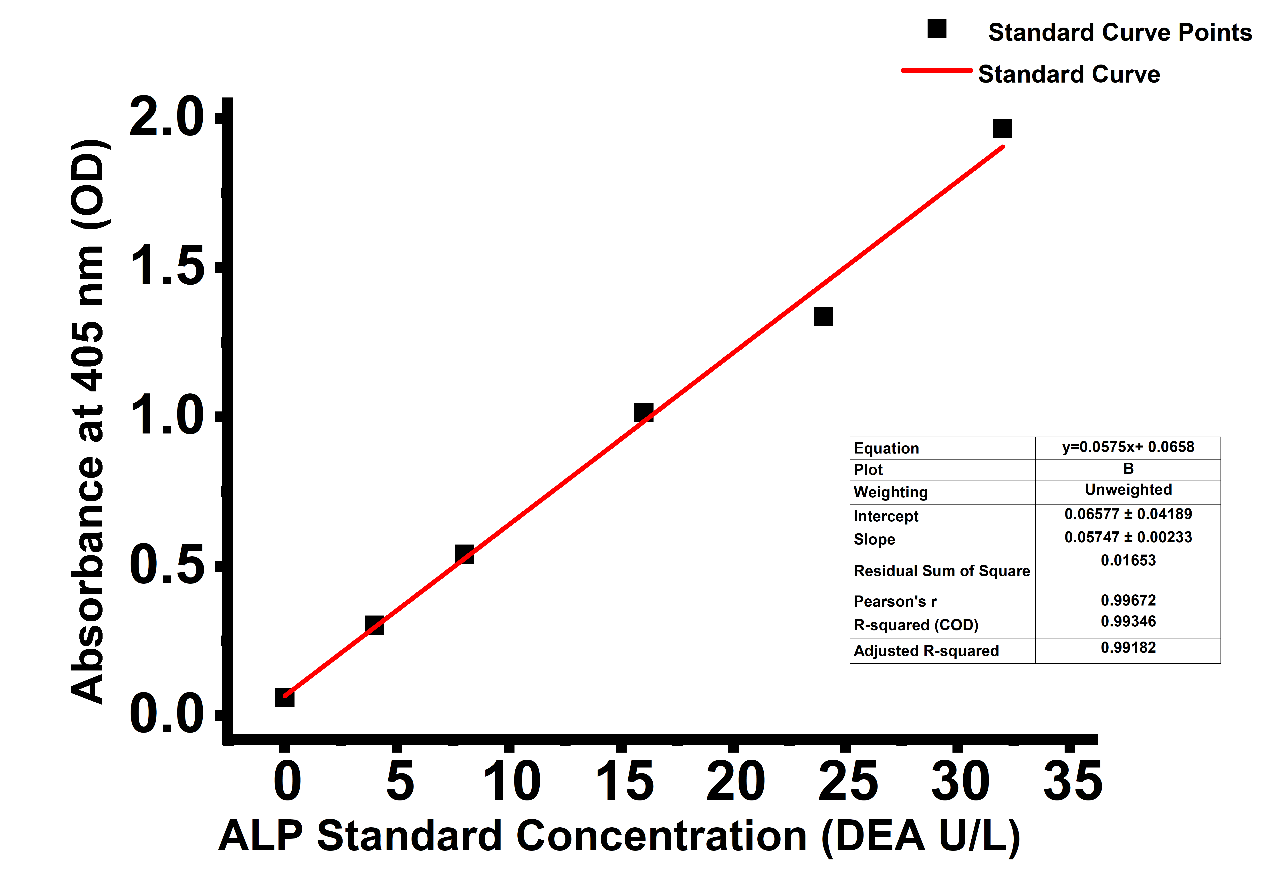


S2 Fig. Standard curve for the quantification of Alkaline Phosphatase (ALP) activity.

The curve was generated using known amounts of *p*-nitrophenol (pNP) (0, 2, 4, 8, 12, 16, 20 nmol/well). Absorbance was measured at 405 nm. The linear regression equation is y = 0.05747x + 0.06577, with a coefficient of determination (R²) of 0.9935, indicating excellent linearity. This curve was used to convert the absorbance readings from cell lysates into absolute amounts of pNP produced, which were subsequently used to calculate the ALP activity values presented in Fig. 4A. Data points represent the mean ± SD (n = 3 independent measurements).
