# Supplementary material for: Aptamer-functionalized stiff hydrogel for enhanced BMSC enrichment and osteogenesis
Source: PLoS One. 2026 Jul 16;21(7):e0353772. doi: 10.1371/journal.pone.0353772 (PMC13374975; doi:10.1371/journal.pone.0353772)
Supplement: S6 Text — (DOCX) [file pone.0353772.s007.docx]

# **S6 Text. Quantitative Real-time PCR (qPCR) Analysis for Osteogenic Gene Expression**

## *S6.1 Purpose*

To quantify mRNA expression levels of osteogenic marker genes (RUNX2 and OCN) in rat BMSCs cultured on different hydrogel substrates.

## *S6.2 Methods Summary*

Total RNA was extracted with RNAprep pure Cell/Bacteria Kit (TIANGEN). cDNA was synthesized from 1 μg RNA with PrimeScript™ RT reagent Kit (Takara). qPCR was performed with TB Green® Premix Ex Taq™ II (Takara) on a CFX Connect™ Real-Time System (Bio-Rad) using gene-specific primers (S1 Text). Primer specificity was confirmed by melt curve analysis (single peak), and amplification efficiency (95%-105%) by standard curves (R² > 0.99) (S3 Fig). Relative gene expression was calculated using the 2^(-ΔΔCt) method (normalized to GAPDH).

## *S6.3 Raw Data Summary and Calculated Relative Expression*

qPCR was performed with n = 3 independent biological experiments (3 technical replicates each). Raw Ct values are provided in the source data file (REALTIME.xlsx). Relative expression (2^(-ΔΔCt)) is summarized in Table S4 (normalized to GAPDH and Control group).

Table S4. Summary of relative gene expression (2^(-ΔΔCt))

| **Biological Replicate** | **Treatment Group** | **RUNX2 (Relative Expression)** | **OCN (Relative Expression)** |
| --- | --- | --- | --- |
| 1 | Control | 1 | 1 |
|  | Sil-MA/SA | 1.413 | 1.493 |
|  | Sil-MA/SA-Apt19s | 2.865 | 2.927 |
| 2 | Control | 1 | 1 |
|  | Sil-MA/SA | 1.394 | 1.602 |
|  | Sil-MA/SA-Apt19s | 2.999 | 2.808 |
| 3 | Control | 1 | 1 |
|  | Sil-MA/SA | 1.301 | 1.484 |
|  | Sil-MA/SA-Apt19s | 2.808 | 2.878 |

## *S6.4 Statistical Analysis*

Relative expression data were analyzed by two-way ANOVA (factors: "Treatment Group" and "Gene") followed by Tukey's HSD post hoc test (GraphPad Prism 9.0). P < 0.05 was considered statistically significant.

S3 Fig. qPCR primer specificity and amplification efficiency validation.

(A) Melt curves for RUNX2, OCN, and GAPDH primers (single peaks confirm specificity). (B) Standard curves for primers (R² > 0.99, amplification efficiency 95%-105% confirm reliability). Data represent mean ± SD (n = 3 independent measurements).
