# Supplementary material for: Aptamer-functionalized stiff hydrogel for enhanced BMSC enrichment and osteogenesis
Source: PLoS One. 2026 Jul 16;21(7):e0353772. doi: 10.1371/journal.pone.0353772 (PMC13374975; doi:10.1371/journal.pone.0353772)
Supplement: S7 Text — (DOCX) [file pone.0353772.s008.docx]

# **S7 Text. Alizarin Red S (ARS) Staining for Mineralization Assessment**

## *S7.1 Cell Culture and Osteogenic Induction*

Rat BMSCs were seeded onto hydrogel substrates (Control, Sil-MA/SA, Sil-MA/SA-Apt19s) and cultured in complete growth medium until 80% confluence. Osteogenic differentiation was induced with osteogenic medium (growth medium + 10 mM β-glycerophosphate + 50 μM L-ascorbic acid + 10 nM dexamethasone), changed every 2-3 days. After 21 days, cells were processed for ARS staining.

## *S7.2 Staining Procedure*

Fixation: Culture medium was aspirated. Cells were washed twice with pre-warmed PBS, then fixed with 4% paraformaldehyde (PFA) for 30 min at room temperature.

Washing: PFA was aspirated. Cells were washed twice with PBS (5 min per wash) to remove excess fixative.

Staining: 500 μL 2% ARS solution (pH 4.1-4.3) was added to each well. Plates were protected from light and incubated at room temperature for 5 min with gentle agitation.

Destaining/Washing: Staining solution was aspirated. Cells were washed 3-4 times with distilled water (5 min per wash) until supernatant was clear.

Microscopy: Stained samples were kept in a minimal volume of PBS. Multiple random fields per sample were imaged with an inverted phase-contrast microscope (Olympus IX73) under bright-field illumination.

## *S7.3 Quantitative Analysis*

After imaging, ARS dye was eluted with 10% (w/v) cetylpyridinium chloride (CPC) solution (30-60 min, room temperature, gentle shaking).

Eluate was transferred to a new 96-well plate, and absorbance was measured at 405 nm.

Absorbance values were normalized to total protein content or cell number of corresponding samples.

## *S7.4 Statistical Analysis*

Data are presented as mean ± SD (n = 3 independent biological experiments). Significance was determined by one-way ANOVA followed by Tukey's post hoc test (GraphPad Prism 9.0). P < 0.05 was considered statistically significant.
